# Late effects of cancer (treatment) and work ability: guidance by managers and professionals

**DOI:** 10.1186/s12889-021-11261-2

**Published:** 2021-06-29

**Authors:** Ingrid G. Boelhouwer, Willemijn Vermeer, Tinka van Vuuren

**Affiliations:** 1 Department of Applied Psychology, Amsterdam University of Applied Sciences, Wibauthuis/Wibautstraat 3b, 1091 GH Amsterdam, The Netherlands; 2 Faculty of Management, Open University of The Netherlands, Heerlen, The Netherlands / Loyalis Knowledge & Consult, Heerlen, The Netherlands

**Keywords:** Cancer, Employment, Job resources, Late effects, Organization, Psychology, Self-employed, Work ability

## Abstract

**Background:**

The prevalence of the group of workers that had a cancer diagnosis in the past is growing. These workers may still be confronted with late effects of cancer (treatment) possibly affecting their work ability. As little is known about the guidance of this group, the aim of this study was to explore the experiences and ideas of managers and professionals about the guidance of these workers in the case of late effects of cancer (treatment). Given the positive associations with work ability of the job resources autonomy, social support by colleagues and an open organisational culture found in several quantitative studies, these job resources were also discussed. Further ideas about the influences of other factors and points of attention in the guidance of this group of workers were explored.

**Methods:**

Semi-structured interviews were conducted with managers (*n* = 11) and professionals (*n* = 47). Data-collection was from November 2019 to June 2020. The data were coded and analysed using directed content analyses.

**Results:**

The late effects of cancer or cancer treatment discussed were physical problems, fatigue, cognitive problems, anxiety for cancer recurrence, and a different view of life. The self-employed have less options for guidance but may struggle with late effects affecting work ability in the same way as the salaried. Late effects may affect work ability and various approaches have been described. Autonomy, social support of colleagues and an open organisational culture were regarded as beneficial. It was indicated that interventions need to be tailor-made and created in dialogue with the worker.

**Conclusions:**

Especially with respect to cognitive problems and fatigue, guidance sometimes turned out to be complicated. In general, the importance of psychological safety to be open about late effects that affect work ability was emphasized. Moreover, it is important to take the perspective of the worker as the starting point and explore the possibilities together with the worker. Autonomy is an important factor in general, and a factor that must always be monitored when adjustments in work are considered. There is a lot of experience, but there are still gaps in knowledge and opportunities for more knowledge sharing.

**Supplementary Information:**

The online version contains supplementary material available at 10.1186/s12889-021-11261-2.

## Background

A vast majority of the working population diagnosed with cancer returns to work. Return to work rates range from 60 to 92% (with a median interval of 2 years) in a review study on data from Mediterranean and Central European countries [1]. Mean rates for return to work in other reviews are 62% [2], 64% [3], and 73% [4]. The long-term survival for common cancers of working age, such as breast cancer, is still increasing and the retirement age is rising in several countries in Europe, resulting in a faster growing prevalence of the group of workers that have had a cancer diagnosis in the past. The first 2 years after cancer diagnosis is an important period in many countries because of legal rules regarding the reintegration process. Therefore, studies among workers with a past cancer diagnosis are mainly focussed on return to work and guidance of these workers in the first 2 years after cancer diagnosis [5–7]. Studies concerning guidance by the employer during these first 2 years describe return to work processes as difficult to manage [8] and offer interventions focussed at communication to enhance return to work [9]. Furthermore, in recent years more attention is paid to guidance by health care workers shortly after diagnosis, regarding work as one of the treatment goals [10], and focus rehabilitation efforts also on employment as an outcome [11].

However, after return to work, workers with a past cancer diagnosis may still be confronted with a range of physical and psychological changes. These changes may be present since the treatment was given and persist on the long term, or changes may appear months or years later at first [12] and continue to influence the lives of those concerned [13]. As a clear distinction between long-term and late effects is not always possible, all these changes in the present study are indicated as late effects in line with the definition of the Dutch Federation of Cancer Patient Organisations [14]. Late effects include, for instance, physical problems [15], fatigue [16–18], or cognitive problems (e.g. problems with concentration, learning and memory) [19].

Late effects of cancer or cancer treatment may affect work ability [20–22]. Work ability refers to one’s ability to be able to achieve expected work goals [23, 24]. When used in qualitative studies work ability may be described as the extent to which the worker physically, as well as mentally, is able to work, now and in the near future. In studies with a quantitative design one or more items of the Work Ability Index (WAI) questionnaire [24] are frequently used to measure work ability [25]. Quantitative studies report that the level of work ability is an indicator for other work outcome measures, for instance for receiving a disability pension [26], absenteeism or early retirement [27] among healthy populations. As the focus of the present study is on experiences and opinions of managers and professionals regarding workers experiencing late effects of cancer (treatment) and their work ability, and the actual and possible guidance offered by the managers and professionals, a qualitative design is used.

Furthermore, the well-established Job Demands-Resources (JD-R) model [28] is used to explore and analyse the guidance offered and explore any further ideas to preserve and enhance the work ability of workers confronted with late effects. In the JD-R model the so-called job demands are regarded as the aspects of the job that require effort. Late effects of cancer and cancer treatment may result in work demands being experienced as heavier. However, on the other hand supporting factors, the so-called job resources, may have a relieving effect. Among healthy populations job resources are positively related to work ability [29], as well as among workers with chronic diseases [30] and among workers with a past cancer diagnosis [25]. Therefore, it is important to explore job resources as targets of interventions in the guidance of workers confronted with late effects of cancer (treatment).

Of course, the possibilities to make use of guidance are not equal across all workers. Salaried workers can make use of guidance within and outside their organisation that is offered by the employer, while the non-salaried, like the self-employed, are in a less favourable situation as they lack these opportunities and they, for instance, cannot consult an occupational physician for free. Besides this, the non-salaried already more often continue working during treatment [31], suggesting their situation in general offers less possibilities to recover. Therefore, this study focuses on the salaried, as well as the non-salaried.

 Several studies on the support of workers with chronic conditions focus on multi- and interdisciplinary guidance [32–34], while this is rarely the case for workers shortly past cancer diagnosis and not at all if the cancer diagnosis is more than 2 years ago, as far as the authors are aware off. However, various professionals do offer guidance to this group of workers because of late effects affecting work ability. In short, the guidance of workers that returned to work, experiencing late effects of cancer (treatment), is an important aspect of the tasks of some managers and many professionals, but a neglected research area.

To summarize, the aim of this study is: 1) to explore the roles, experiences, possibilities and ideas that managers and professionals have regarding the guidance of workers confronted with late effects of cancer (treatment), 2) to explore the role of job resources in reducing the possible impact of late effects on work ability, and 3) the ideas about other opportunities in the guidance of this group of workers. This knowledge can contribute to an understanding of what is possible in the context of work to preserve work ability and to prevent relapse among workers experiencing late effects of cancer or cancer treatment that may affect work ability.

The structure of the article is as follows. First our methodology will be described. Second, the results section of this study starts with the information regarding the characteristics of the group of interviewees, that is managers and professionals. Then follows the description of the information provided regarding their experience with workers confronted with late effects of cancer (treatment) and general information about the possible guidance given. Then, the results on the role of job resources as targets of intervention will be addressed. Further, additional ideas related to the guidance of this group of workers will be reported. Finally, our conclusions and discussion will offer some important points of discussion of relevance regarding the guidance of workers confronted with late effects of cancer (treatment) affecting work ability.

## Methods

### Participants and recruitment procedures

Semi-structured interviews were conducted with managers and professionals active in the field of guidance and support of the working population. Managers could be active at different organisational levels. Professionals were active in human resource management, in case management, or as an occupational physician (with or without a specialist additional training as a consultant oncology, in Dutch a so-called BACO), an occupational health expert, nurse specialized in cancer working in an organisational context, reintegration consultant, or coach. During the recruitment process, it was decided to also include occupational therapists, an artistic therapist, a music therapist, and oncological physiotherapists, because these professionals also guide workers more than 2 years after cancer diagnosis regarding their occupational functioning.

Recruitment was done by e-mail. Several professional associations, non-profit and profit companies, stakeholders, managers, and professionals in the researchers’ networks were approached to see whether there was interest in participating. A letter with information was used to inform the participants about the aim of the study, the data collection, data storage and analyses. It was explained that questions would be asked about their experience with workers who have had a cancer diagnosis in the past and who have been treated for cancer. Furthermore, it was stated the interviews concerned their ideas about the possibilities in the approach and advice in practice. No reward was promised. A form was used to ask the participants for their informed consent.

The Research Ethics Committee (cETO) of the Open University of the Netherlands assessed the ethical acceptability of the study and agreed with the study design and method (reference cETO: U/2019/07620/MQF).

### Interview topics and data collection

The interview guide was developed for this study and has not been published previously elsewhere. An English language version of the interview guide is available as a supplementary file. See Additional file 1, Interview Guide.

Draft versions of the interview guide were pre-tested by the first author and the research assistants. The topics (and related open questions) of the interview guide can roughly be grouped as follows: 1) late effects of cancer or cancer treatment, 2) impact of late effects on work ability, 3) possibilities in the context of work to alleviate the possible impact of late effects, with an a priori focus on autonomy, social support by colleagues, and the role of the organisational culture. The questioning offered the freedom for the interviewees to decide the extent to which they could address the topics.

The interviews were face-to-face, remotely by video calling or by telephone. The data collection took place from November 2019 up to June 2020. In the middle of this period, in March 2020, measures were taken because of the COVID-19 pandemic. From then on, all interviews were conducted remotely. The interviews took 30 to 60 min. All interviews were audio taped. A draft of the interview report was sent to the interviewee by e-mail by the first author and any deletions or adjustments could be made by the interviewee and sent to the researcher by a reply. After the interview report was approved by the interviewee, it was made anonymous and given a unique code. The final interview reports were imported into MAXQDA 2020. See Additional file 2, Flow Chart Research Methodology.

### Data analyses

The data were coded and analysed using directed content analyses [35], as this method allows to use existing theory or prior research to develop a coding scheme before the start and to revise and refine the code scheme during coding. Furthermore, it was taken into account that in addition to the a priori themes, additional themes within the objective of the study may emerge. Consequently, each of the three research assistants coded three interviews of a sample of nine interviews covering all occupational interviewee roles. The coding was discussed with the first author and the other research assistants to make sure all relevant information was coded. New codes that emerged from the data were discussed and, if relevant, added. Any discrepancies were resolved through negotiated consensus. Subsequently, the remaining 49 interviews were divided between the three research assistants for a first coding. To support reliability, the first coding was done by a research assistant that was not present at the interview in question. The first author checked all coded interviews and brought forward any discrepant coding to be able to resolve these by negotiated consensus. On all coding agreement was reached.

## Results

### Results: participants and their professional contact with workers more than 2 years past cancer diagnosis

An overview of all participants, offering information regarding their characteristics (gender, professional roles, and organisational context) is presented in Table 1. Also, the individual codes are presented, with the letter indicating the (primary) professional role. See Table 1, Participating managers (*N* = 11) and professionals (*N* = 47): codes, organizational context, any other professional role(s) and gender.

**Table 1:** Participating managers (*N* = 11) and professionals (*N* = 47): codes, organizational context, any other professional role(s) and gender

**Code participant**	**Managers (*n*** **= 11)**	**Organizational context**	**Other professional role(s)**	**Gender**
M1	Manager and professor	University of applied sciences		F
M2	Manager	Information and Communication Technology company		M
M3	Manager	Information and Communication Technology company		M
M4	Manager	Governmental organization		F
M5	Manager	University of applied sciences		F
M6	Manager	Municipal service	Previous: human resource management	M
M7	CEO	Commercial company		F
M8	Manager	Centre of physiotherapy	Additional: physiotherapist	M
M9	Manager	University of applied sciences	Self-employed coach	F
M10	Manager and coach	Welfare organization		F
M11	Manager	Municipal service	Self-employed coach	F
**Code participant**	**Professionals (*n*** **= 47)**	**Organizational context**	**Other professional role(s)**	**Gender**
AT1	Art therapist	One-person bureau		F
CO1	Coach	University of applied sciences		F
CO2	Coach	University		F
CO3	Coach	Coaching bureau (specialised in major life events)		F
CO4	Coach	Coaching bureau (specialised in major life events)		F
CO5	Coach	Coaching bureau	Previous: physiotherapist	F
CO6	Coach	Coaching bureau (specialised among others in cancer)		M
CO7	Coach and trainer	One-person coaching bureau (specialised in cancer)		F
CM1	Case manager sickness absence and employability	Telecommunication company		F
CM2	Case manager sickness absence and employability	Telecommunication company		F
CM3	Case manager	Non-profit psycho-oncological drop-in-centre		F
HR1	Human resource management	University of applied sciences		F
HR2	Human resource management	Bureau for human resource management		F
HR3	Human resource management	University of applied sciences		F
HR4	Human resource management	University of applied sciences		F
HR5	Human resource management	University of applied sciences		F
HR6	Human resource management	Health insurance company	Additional: career coach and trainer	F
HR7	Human resource management, specialized in reintegration and career counselling	Hospital		F
MT1	Music therapist	One-person bureau		F
N1	Nurse specialized in cancer	Transport company (health and safety service department)		F
OH1	Occupational health expert	One-person bureau	Additional: coach and therapist	F
OH2	Occupational health expert	Social security organization		F
OH3	Occupational health expert	One-person bureau		F
OH4	Occupational health expert	One-person bureau		F
OH5	Occupational health expert	Police organization and self-employed		F
OH6	Occupational health expert	Police organization		F
OH7	Occupational health expert	One-person bureau	Additional: coach and trainer	F
OP1	Occupational physician	University and academic hospital (health and safety service department)		F
OP2	Occupational physician	University (health and safety service department)		F
OP3	Occupational physician	Transport company (health and safety service department)		M
OP4	Occupational physician	Financial company (health and safety service department)		F
OPB1	Occupational physician consultant oncology (BACO)	Organizational consultancy company	Additional: coach, mediator, trainer	M
OPB2	Occupational physician consultant oncology (BACO)	Organization of occupational consultancy		F
OPB3	Occupational physician consultant oncology (BACO)	Hospital and independent health and safety service company		F
OPB4	Occupational physician consultant oncology (BACO)	University (health and safety service department)		F
OPB5	Occupational physician consultant oncology (BACO)	Academic hospital (health and safety service department)		F
OPT1	Oncological physiotherapist	Centre of physiotherapy		F
OPT2	Oncological physiotherapist	Hospital (rehabilitation department)		F
OT1	Occupational therapist and coach	Reintegration bureau		F
OT2	Occupational therapist and coach	Reintegration bureau		F
OT3	Occupational therapist	Organization for occupational therapy		M
OT4	Occupational therapist	One-person bureau		F
RB1	Reintegration consultant	Reintegration bureau (specialised in cancer)	Self-employed career counsellor	F
RB2	Reintegration consultant	Reintegration bureau (specialised in cancer)		F
RB3	Coach and managing director	Reintegration bureau (specialised in cancer and serious diseases)		F
RB4	Coach	Reintegration bureau (specialised in cancer and serious diseases)		F
RB5	Coach and managing director	Reintegration bureau (specialised in cancer and chronic diseases)		M

*F* female, *M* male

Of the 58 interviewees 19% (*n* = 11) had a managerial role and 81% (*n* = 47) had a professional role at the time of the interview. The majority (84%) of the 58 interviewees was female; this concerned 64% (*n* = 7) of the managers and 89% (*n* = 42) of the professionals. Not mentioned in the table, is the fact that of those interviewed 26% (*n* = 15) spontaneously mentioned a personal cancer diagnosis in the past during the interview.

Thirty-six per cent (*n* = 4) of the managers also had experience with one of the professionals roles, for instance as self-employed coach (M9, M11) or as psychotherapist (M8). Eleven per cent (*n* = 5) of the 47 professionals had more than one professional role at the time of the interview, for instance an occupational health expert also working as a trainer (OH7). Furthermore, 17% (*n* = 8) of the professionals worked their full working time as self-employed in their one-person business (AT1, CO7, MT1, OH1, OH3, OH4, OH7, OT4).

Almost all interviewees had (to various degrees) professional experience with workers more than 2 years after cancer diagnosis. During the interview, two of the occupational therapists (OH1, OH2) reported that their professional experience was focused on the first 2 years after cancer diagnosis, but they did also have experience with the group more than 2 years past cancer diagnosis, for instance because of voluntary work in a walk-in centre. Furthermore, one manager and one coach did not have the professional experience with workers more than 2 years past cancer diagnosis, but a strong affinity with the issue because of experience with the issue outside the professional role (M4) or experience with workers with complaints due to chronic diseases (CO1). One case manager (CM3) worked for a large non-profit psycho-oncological walk-in-centre. This case manager offers advice and guidance on absenteeism and reintegration for workers with a cancer diagnosis that visit the walk-in-centre, and also has contact with workers more than 2 years after diagnosis. One manager (M7) had extensive managerial experience, and now was CEO. To summarize, not all interviewees had (recent) experience with workers more than 2 years past cancer diagnosis in the workplace but these interviewees were included in the data-analyses because of their relevant experience in previous or other (work) contexts with this issue.

Within organisations, the managers, and within some companies a specialized case manager (CM1 and CM2) or a specialized human resource manager (HR7), had the role to guide the workers with a past cancer diagnosis during the reintegration process and thereafter. The two specialized case managers were part of the human resource management department. However, in general, those active in human resource management in most companies and organisations acted on distance from the workplace by advising the managers, and the manager was regarded as responsible for the contact with the employees, while human resource management was only to be involved in the case of a complex situation. Consequently, the direct contact of human resource management with workers with a past cancer diagnosis was reported to be limited.

However, workers confronted with late effects of cancer or cancer treatment also contact various professionals of their own choice for help or guidance. The contact with professionals not related to the organisation or company of their employer may primarily concern work related issues, but the primary reason for consultation may also be the coping with one of the many other aspects of cancer and cancer diagnosis affecting the person. However, also in the case of the latter, functioning in work may be addressed as well. An example was offered by a physiotherapist, not only focusing on movement therapy regarding the physical late effects of cancer treatment, but also focusing on work issues and mental aspects of recovery (OPT1).

During the interviews, it appeared that some professionals tend to work together regularly. This concerns predominantly occupational physicians working together with occupational health experts and occupational therapists. An occupational health expert (OH7) told that it differs per assignment with whom will be worked together and sometimes contact is made with the occupational physician, so that they do not get in each other’s way but strengthen each other. Occupational physicians reported to have also direct contact with managers and human resource management depending on the situation. Also, several times it was mentioned by different professionals that advice regarding the adjustment in working hours is the responsibility of the occupational physician. Furthermore, the occupational physicians with a specialist additional training as a consultant oncology (BACO), can also be available to be consulted by the occupational physicians without this additional training. The situation regarding collaboration between different professionals other than the above-mentioned concerning workers with late effects after cancer or cancer treatment seems less obvious.

To summarize, the professional field regarding the guidance of workers with late effects of cancer (treatment) appears to be very broad and varied, with a collaboration between certain professionals within certain networks related to work organisations, however no clear view on collaboration with professionals outside these networks can be offered from the present study.

### Results: experience regarding late effects of cancer or cancer treatment and ideas concerning guidance

Various late effects of cancer or cancer treatment were discussed. The prepared interview questions were a priori about possible experiences with physical late effects, fatigue, cognitive problems, and there was an open question to ask for other late effects experienced by workers past cancer diagnosis. As a result, two other late effects emerged during several interviews, namely the fear of cancer recurrence and a different approach to life.

#### Physical problems

In 36% (*n* = 21) of the interviews, physical late effects were discussed as something experienced among workers. Examples of physical late effects are the effects of surgery (such as lymphedema and difficulty with arm movements), neuropathy caused by chemotherapy, pain in the joints because of treatment with endocrine effects, heart problems, and a decreased resistance to common diseases was also mentioned. Physical problems may impair the ability to keep the job or to preserve work ability. The extent to which this is the case depends on the degree to which the work is physically demanding.

Possible solutions depend highly on the situation and examples were given about solutions targeting at the effects of the specific physical late effect and resulting in practical adjustments in the work task, work processes and the work environment, for instance working in a couple with someone else who can handle certain too heavy physical tasks.

#### Fatigue

In 79% (*n* = 46) of the interviews, fatigue was discussed as a late effect experienced among workers. Ideas about causes and ways of coping with this problem were discussed in a number of these interviews. Fatigue is indicated as a common late effect of cancer treatment. This late effect is reported as a possible cause of relapse after the worker had already been reintegrated into work (OPB4). By several interviewees fatigue is also indicated differently, for instance in terms of problems with energy or lack of vitality. Workers may say: “I don’t know why, but I can’t.” (OPB1). The interviewees presented various examples of this late effect among workers past cancer diagnosis. Fatigue after cancer treatment is also described as unpredictable and uncontrollable (HR6, RB1), causing a lot of frustration in the worker, of a chronic nature in many cases (OPT2), and as something that takes time to recover from (OH5). It is stated that those with a cancer history can lose energy quite suddenly (HR6). An occupational health expert (OH1) considers its clinical presentation comparable with fatigue after non-congenital brain injury. Also, some interviewees distinguish physical and mental or emotional fatigue (M9, OH1, OT3, OPT1).

Furthermore, it is reported that fatigue may have various causes, like the processing of getting cancer, mentally fighting with the situation, cognitive problems, not being used to activation anymore, stimulus sensitivity and a working environment that has not yet been adjusted. Furthermore, the late effect fatigue is not always understood by others (OPB1). After cancer treatments fatigue may manifest at all ages, also at younger age (OPB5). It is also brought forward that with advancing age it is difficult to be certain that the fatigue is due to the cancer treatments, and not to normal aging (HR4, OH3, RB2) or normal menopausal complaints (RB2, RB4). In addition, one of the occupational physicians indicates that the clinical presentation of fatigue in workers past cancer diagnosis is not different from fatigue in the elderly workers with chronic diseases (OP2). However, workers with a chronic disease are also said to be able to more easily trace back the cause of the fatigue (OP4). The group of cancer patients is also reported to be different in the extent to which they lose energy and at the same time want to be optimistic. Furthermore, it is underestimated how much recovery time is required (OPB2).

Workers with a cancer history are mentioned to be a group with a high motivation for work by several of the interviewees, and therefore at risk for running up against their own limits (OH6). Several interviewees explicitly mention that fatigue affects job performance or work ability, and it is also stated that this group of workers often gives job performance a higher priority than activities outside work. It is a process of nibbling on the social life of the person in question and give work a higher priority than the home situation or family (CO7, OPB1, OPB4, OH3). So, then work succeeds, but at home the worker is exhausted and unable to socialize, exercise or go shopping. “Life is more than work alone”, and the different components should get attention in combination (OT3, RB1, RB3). One of the coaches indicates that these people need more time to use their “default mode network” to create the right balance between “doing” and “being” (CO2).

It is also mentioned that some workers past cancer diagnosis develop burnout complaints (HR6, OPB1, OPB4), and then it can be difficult to distinguish burnout complaints from fatigue as a result of cancer treatment, and it is unclear how these problems are possibly related to each other (OPT2). Furthermore, fatigue or a lack of energy is explicitly regarded as associated with cognitive problems by various professionals (OH6, OH7, OPB1, OPT2, OT3, RB5), and some interviewees formulated ideas about reciprocal causality, in other words, the idea that fatigue causes cognitive problems and that cognitive problems cause fatigue. A more implicit remark concerning this issue is that reduced vitality is also expressed in a loss of sharpness and overview (CO5).

Regarding fatigue, guidance is given by professionals by mapping out what type of fatigue it concerns, what maintains the fatigue, and how personal limits and the needed recovery time can be monitored, and how a good work-life balance can be achieved.

#### Cognitive problems

In 60% (*n* = 35) of the interviews cognitive problems were discussed in depth, as this topic had been raised in the contacts between the worker and the interviewee. The interviewees described cognitive problems as being unable to concentrate or switch attention. Also, problems with multi-tasking, memory problems, problems with working memory, sensitivity to stimuli and to disruptions during a task are reported. Examples are workers having trouble reading long e-mails, having difficulty maintaining concentration during a meeting or while working in an open space office, or having difficulty with further education. Important differences between workers were reported as well. For example, some of them experience hardly any or no cognitive problems, especially when no radiation or chemotherapy has taken place (RB1). However, the cognitive problems may not be visible at first glance but can be observed in practical functioning (OT2), or even only during neuropsychological testing. This invisibility of cognitive problems can be quite dangerous, for example, if people are no longer fast enough to press an emergency button (N1). Some tasks are kept away from workers with cognitive problems, such as distributing medication among patients in health care settings (HR7). In this regard, one interviewee said that a rule within the organisation is as follows: “We work safely, or we don’t work” (CM1). Over time, cognitive problems may decrease (OPB2, OPT2), but it is also possible that people can no longer cope with the work tasks or the job (OPB4).

It is also stated that it is important that the employee tells the manager about any cognitive problems (M1). One interviewee indicates that a certain organisational culture that offers the freedom to share these problems is essential (M10). Human resource managers in general do not hear about cognitive problems from the employees in concern themselves, but from the managers. However, it is mentioned by a human resource manager that it is important to know that there is a problem that is regarded as medical in nature because that knowledge affects the content of the discussion with the employee (HR1).

In a number of these interviews also ideas about ways of coping with cognitive problems are discussed. Some of the interviewees bring up that they know of online cognitive training programs. However, an occupational physician indicates that the effect of cognitive training is reported to be limited in the practice of work. Furthermore, a coach indicates that their reintegration bureau offers a training to improve working memory, and that the clients report improvement. Cognitive rehabilitation can also be offered as a part of occupational therapy. Practical solutions found within the workplace are reported as well, for instance offering the opportunity to withdraw of advising to do tasks one by one (OP1). However, one of the case managers expresses the need for knowledge about how to adapt specific work tasks to a less demanding cognitive level.

#### Fear of cancer recurrence

This additional topic was brought up during 22% (*n* = 13) of the interviews. This fear may concern the fear of getting another cancer diagnosis or the fear of getting metastases of the cancer diagnosed and treated in the past. Managers, as well as different professionals, have observed this late effect among workers past cancer diagnosis. “This fear is very tiring”, a manager indicates (M10). Some of the interviewees stated that every person that is curatively treated for cancer and hopefully has survived cancer, may experience some level of fear of cancer recurrence, although there are individual differences in vulnerability (CO4, OH7, RB5). Fear of cancer recurrence can be triggered for instance by severe life events (M9), hearing somebody else had a recurrence of cancer or metastases (AT1, RB5), medical checks (HR1, RB5), minor physical problems (OPB3), a new threat like COVID-19 (CO7), or talking about cancer in general (HR1). How one will act out of fear also depends strongly on how one was guided shortly after diagnosis (CO3, CO4). One interviewee expresses the possibility, that the fear of cancer recurrence indirectly affects mental resilience, which may lead to other complaints that are not directly linked to the diagnosis of cancer (M11).

It is mentioned to be important to be aware this fear may be a problem sometimes, so some supporting attention could be given in the work environment.

#### A different view to life

Another long-lasting effect of a past cancer diagnosis that is brought forward by some interviewees concerns a different view of the future and rethinking the approach to life. This additional topic is discussed in 9% (*n* = 5) of the interviews in depth, and in another 5% (*n* = 3) of the interviews it is indicated that people can feel changed on a personal level. Several interviewees have a strong impression that cancer makes people think more consciously about work. Because of what they experienced people make different choices. They can look at the future differently (CM1). With cancer, the question arises “What am I doing, and do I really want that?” (AT1). A salaried person, in a high position, started to reflect on his work situation after the diagnosis, as he felt confronted with the finiteness of life. Another example is a manager, who started to experience a higher appreciation of activities other than work. Past cancer diagnoses, workers place much more demands on their work, and they want their work to be meaningful (HR7). They may make the choice to do a training or to look for other work in the organisation or to work less hours (OP4). Workers may also experience they changed as a person. “Becoming the old “I“ again is not possible.” (RB3). Two coaches, specialised in major life events (CO3, CO4), also indicated that after a period of suffering people can experience ‘post-traumatic growth’ [36], and that this also can be seen among workers that had a cancer diagnosis. Changes on a personal level may affect the experience of work and choices regarding work. One interviewee more generally stated that it is important to focus on someone’s motivations and needs (CO5), and a cancer diagnosis is brought forward as a cause resulting in the need to do this.

Guidance in the case of fear of cancer recurrence or when the worker is rethinking his or her approach to life, mainly lies with professionals who are not affiliated with an employer. This guidance takes place outside the context of the work.

To summarize, the late effects discussed were physical problems, fatigue, cognitive problems, anxiety for cancer recurrence, and a different approach to life. Both managers and professionals report that late effects may affect work ability. Also, during the interviews, some professionals indicate that it is not always known or accepted that certain complaints are late effects of cancer treatments. Furthermore, it is also suggested that in the case a worker is aware of late effects, this may not always be shared with others in the context of work (HR4). It may be a taboo to tell about late effects (OT1) or the worker may even deny the complaints. As a result, it is possible an employee calls in sick due to late effects, which are not known in the work environment (HR6).

### Results: experience and ideas regarding the role of job resources in the guidance of workers

As late effects of cancer treatment may still play a role in the long run, it is important to know what managers and professionals think about the influence of certain job resources on the work ability of this population workers. In this study the focus is a priori on the job resources 1) autonomy, 2) social support by colleagues, and 3) an open organisational culture.

#### Autonomy

In 48% (*n* = 28) of the interviews autonomy is discussed in depth and it is stated many times that autonomy is important to enhance work ability among all workers. It is also stated that autonomy is important not only for the work ability of workers who experienced cancer diagnosis and treatments, but for all people who have experienced a serious situation (OP3). However, an occupational therapist indicates that autonomy can be decreased because of the cancer treatments (OT1), and a personal difficulty with taking autonomy is one of the reasons of searching professional support (MT1). When someone does not have an understanding manager, the feeling of impairment of autonomy and competence persists (OH1). Furthermore, when people feel that they have little to say in a company, they also take less autonomy (OT4). It is noted several times that the degree of possible autonomy in the work situation depends on the assignment and the extent to which the work content allows for variation (M2, M3, M11, N1, OH1). The self-employed have more possibilities regarding autonomy (OH7), as well as the employed in a higher position (OH4, OPB4). However, this can also work against someone, because these workers often prioritize work over their own capabilities and needs (OH4).

There are different opinions on the issue regarding any possible difference on the importance of autonomy for work ability between workers with and workers without a past cancer diagnosis; some think autonomy is more important among workers past cancer diagnosis and others think autonomy is equally important among both populations. Several examples are brought forward how autonomy can be stimulated to cope with late effects of cancer treatment, like workers themselves deciding to start working at a later time, schedule the working hours, change the work planning, take more breaks if needed, adjust tasks, have possibilities to choose a different work environment (quiet and no open space office), having an opportunity to meditate, or decide to work from home. In large companies, precedent action may be feared if one person is offered something and another is not. Furthermore, possibly the COVID-19 pandemic is a trigger for more autonomy as working from home is more accepted (HR4).

Also, it is important to consider what the worker can handle at work (HR7) and the worker needs to know his or her limits (HR6). However, some warn that a worker should not have a job below their intellectual level, as this takes a lot of energy (CM3), causes under-stimulation (CO5) and the new job may also have fewer job control options (M10). Therefore, switching to a job at a lower function level can be a pitfall (CO5, M10).

In several interviews it is stated that work adjustments must be tailor-made, and the result of a dialogue between the worker and the employer. It is important not to talk about the employee, but talk with the employee, otherwise the employee loses control (HR7). The workers are stimulated to think and communicate about directions for solutions (OPT1, OT3) and their needs (CM1). Finding solutions may require flexibility and creativity from both sides (OPB5). Several managers and professionals pointed out that self-leadership can be important. However, self-leadership may be difficult for some (OP2) and to come up with solutions is difficult for a worker in a such a situation (OT1). Managers indicate that managers should take the lead (M8) and be sensitive in order to estimate the extent to which an employee can take self-leadership. If necessary, a manager should offer external coaching for this (M1). Furthermore, self-leadership can also mean that a worker does not want to talk about late effects (HR7). Also, a therapist indicates that difficulties with autonomy can be a reason for therapy (OT1). Moreover, an occupational physician indicated that with the current so-called ‘self-leadership approach’ the various responsibilities or actions need to be clear and this is not always the case (OPB3). Furthermore, the organisational system itself also must facilitate self-leadership, otherwise it is too easy to point to the employee (M7).

#### Social support by colleagues

In 55% (*n* = 32) of the interviews social support by colleagues is discussed in depth. Colleagues often do not realize that there may be late effects of the treatments for a longer time after a cancer diagnosis, nor that these effects can sometimes occur quite suddenly after many years. Immediately after diagnosis there is a lot of support from colleagues, however several interviewees report that this support decreases after returning to work; the longer it has been, the less understanding (HR2). Late effects are often no longer a topic of discussion, while colleagues can support a worker confronted with late effects enormously by thinking about possibilities to cope with these problems in the context of work, with a positive effect for work ability. That is why it was noted by two occupational physicians (OPB1, OPB2) to be important that colleagues know about certain late effects that are not observable, such as a low energy level. It is important that the work environment knows what it means to have a colleague with or after cancer (CO6). Colleagues should also know how someone is feeling mentally. Several interviewees indicate, that when something happens, the support one gets from colleagues depends on the extent to which the relationship between the worker and the colleagues was already good before the cancer diagnosis. An interviewee stresses the importance of a feeling of inclusion of workers (CO6). Furthermore, it is brought forward that the behaviour of a manager works as an example behaviour. The moment a manager ‘ignores’ or ‘writes off’ someone, the team does the same (HR1).

#### Open organisational culture

In 28% (*n* = 16) of the interviews the organisational culture is discussed in relation to the issue of possible late effects and work ability. Several interviewees indicate that openness between the manager and the employee is important and therefore the psychological safety to be able to share issues. This is perceived to be connected to the organisational culture in general, and hence also experienced to be reflected in the approach of human resource management. Examples of organisations with less openness to discuss late effects are organisations with more men than women (M6, OH2). A kind of family culture within an organisation works positively and gives more openness (M9, M11). Furthermore, a more competitive culture is regarded as less psychologically safe (M7). Especially in the commercial business there may be judgments about workers with cancer. Some people with cancer choose not to tell because of the judgments they may encounter in the workplace (CO2). Workers may not share that they cannot cope with the workload anymore because they fear to lose their job (HR2, OH4) or managerial position (HR2). However, in some organisations psychological safety and the freedom to share problems is an explicit goal to focus on (CM1).

### Results: important general points regarding the guidance of workers more than 2 years after cancer diagnosis

Apart from the ideas regarding guidance specifically in the situation of a particular late effect of cancer or cancer treatment or in relation to a specific job resource, several topics emerged that are relevant in various situations. These topics concern 1) the communication with employees, 2) the monitoring of employees beyond 2 years after cancer diagnosis and return to work, 3) the special position of human resource management within organisations, and 4) experiential knowledge with cancer.

First, several interviewees emphasize that contact with workers confronted with a cancer diagnosis starts as short as possible after diagnosis. Then immediate attention and guidance is needed and contact at a later stage builds on that. The ideas of the interviewees about the division of roles in the initiative regarding the communication in the workplace 2 years or more after diagnosis differ somewhat. For instance, some interviewees indicate that a manager should not have to make inquiries with a certain regularity, because in the regular contact between a manager and an employee any matters should come up naturally. Good contact therefore is stated to be essential. However, it is also suggested that a manager should occasionally check how someone is doing (HR6), however it is difficult to determine the correct level of attention (HR5). The latter is certainly difficult, when late effects are not visible, and an employee may not like too much attention (HR5). Also, several of the interviewed coaches indicate that it is very important that a manager keeps checking how things are going (especially if there is a medical check-up scheduled), shows empathy and also takes into account what someone needs or does not need, does not fill in anything for the employee and listens well and open to the need of the worker in concern. Several occupational physicians consultant oncology (BACO) indicate that it is important that managers stay on top, plan with the employee, including, for example, who is taking the initiative. However, when a new manager is appointed, things can go wrong. He or she has not experienced the illness period of the employee in concern and sees the employee without visible illness (OPB1). One interviewee indicates that employees also have a role in this and could inform their employer about their cancer history (HR2). However, the remark is also made, that even the worker may not know that the complaints affecting work are the late effect of the cancer treatment.

The second issue is related to communication and is about the possible more systematic monitoring of employees with a past cancer diagnosis. Some interviewees indicate that it is important to always keep a finger on the pulse also on the longer term. It is also suggested that it could be important that occupational physicians see a worker after cancer once a year or 2 years if the employee needs this. Workers could be monitored in the context of relapse prevention (OPB4). However, for privacy reasons, the initiative for this must lie with the employee (M10). Furthermore, an occupational physician indicates that a manager does not remain responsible. For the long term, it is better to see how employees become stronger and that they are aware of their own limits, balance, motives, and goals (OPB2). Furthermore, one of the specialised case managers working for human resource management within a large company, points out that, for example, a follow-up path within organisations could be created. This follow-up-path ensures that workers who have had cancer remain under the attention, they can always ask for help and the organisation knows how they are doing (CM2). Beside all this, one should not forget that there are also employees who do not experience late effects or do not want to discuss their cancer history.

The third issue is the special position of human resource management within organisations. The role of human resource management is to offer help in bridging a possible gap between the manager and the employee (HR3). This can be about communication, but also can concern thorough investigation of the situation: “What is the question behind the question?” (HR1). So, the position of human resource management is regarded as important, however some remarks were made. Several professionals said that human resource management is somewhat more distant and (too much) aimed at regulations. It is also noted by several interviewees that it would be good if human resource management, as well as managers, knew more about cancer and work in general and about the possible late effects after cancer treatment in specific. It is mentioned also that managers seem to have trouble with linking the problems with work to a previous cancer diagnosis (HR3). However, on the other hand, it is also said that it is important that a manager should take on a managerial role and not have a medical conversation. The latter is a pitfall that can occur in medical settings, for example in hospitals, because the managers are often physicians or nurses (OP2). Human resource management is in the position to detect points for improvement in the communication or a need for more information and take action. Two interviewees working in a large company within the human resources department as case managers sickness absence and employability (namely CM1 and CM2) describe how they guide workers with late effects of cancer treatments and their managers. They indicate that, although the knowledge about cancer and late effects is crucial, the guidance should be focussed on the work situation. Therefore, these case managers actively build up knowledge and experience with employees that had a cancer diagnosis in the past. “When a manager and an employee engage in a discussion, much more is possible than you think. This must be stimulated and driven by human resource management”, a manager also states (M7).

Fourth, another factor that is brought forward is the personal experience with cancer. An occupational physician with a personal experience with cancer treatments explicitly reported that she understood the experience of cognitive effects shortly after treatment much better since having these treatments herself. Therefore, it is important to realize that at least 26% of interviewees had cancer themselves. However, the effect of experiential knowledge in general is unknown and was no explicit part of the interview topics. Nevertheless, the results of this study indicate that experiential knowledge of managers or professionals may be an additional source of knowledge that can influence the guidance of workers.

## Discussion

This qualitative study among managers and professionals regarding their experiences with and ideas regarding the guidance of workers with possible late-effects of cancer treatments made clear that late effects still may affect work ability of these workers. Studies on this issue are scarce, but similar results have been reported before.

Several previous studies quantitatively indicated that physical complaints after cancer treatment continue to show associations with lower work ability beyond the first 2 years after cancer diagnosis [15, 20, 37–40]. Interviewees in our study described that the impact of physical late effects depend on the type of physical complaint and the type of work tasks. Therefore, the guidance in case of physical problems was always seen as tailor-made, to be developed in consultation with the worker.

Fatigue was a late effect that many of the interviewees had observed impairing work ability among workers past cancer diagnosis and treatment. The association of fatigue with lower work ability has also been established in several quantitative studies [15, 21, 39]. The interviewed professionals in the present study reported that fatigue as a late effect of cancer (treatment) may be a complicated issue to handle, among other things due to the unpredictability and because different forms and causes can be identified. It is reported before that fatigue may be caused and sustained by a variety of factors from different angles, not only by treatment side effects and psychosocial factors, but also by direct effects of cancer and tumour burden, comorbid medical conditions, and exacerbating comorbid symptoms [41]. The options for guidance by managers can be limited here, but professionals can provide guidance to the employee and advice to the manager.

Cognitive problems were regarded as a potential effect on the performance of work tasks needing for concentration and divided attention. This is in line with other qualitative studies [42] and quantitative studies, that report negative associations of cognitive complaints among workers past cancer diagnosis with work ability [15, 22, 43]. An important point that was mentioned in the interviews was that the invisibility of this late effect makes it extremely important that a worker can be open about this and that there is adequate communication to explore possible solutions. The concrete ideas regarding the guidance of workers with cognitive complaints were predominantly focused on the need to clearly identify whether someone could still perform certain (risky) tasks, have the possibility to work in environments with fewer stimuli and plan work schedules to have moments to rest. However, it was also expressed that there is a need for more knowledge about possible adjustments at task level in the case of cognitive complaints. Cognitive strategy training is a focus of research in the area of rehabilitation [44], but was not brought up in the interviews within companies and organisations. Possibly, cognitive strategy training only reaches the workplace of workers with late cognitive problems if they receive guidance from a specialized professional.

In the interviews it also emerged that cognitive problems, fatigue, problems with energy and vitality, and possibly also burnout complaints, are regarded to be related and part of complex interrelations. The relationship of fatigue and cognitive problems has emerged in several studies, such as in a longitudinal study among working cancer patients during 18 months after return to work [45]. Furthermore, exhaustion is part of the construct of burnout complaints [46] and during the interviews it was indicated that burnout complaints can be difficult to distinguish from a lack of vitality or fatigue. The possibility that cancer-related cognitive problems may be mistakenly interpreted as burnout symptoms was also reported in a recent study using focus groups with survivors and professionals [47]. To our knowledge no quantitative studies on possible interrelations of fatigue with burnout complaints are available yet, however we think it is conceivable that late effects of cancer (treatment) can maintain fatigue and trigger burnout complaints. Regarding solutions to such interrelated complaints that affect work ability, it is therefore important to properly unravel causes and consequences, urging an interdisciplinary approach using both work and organisational psychology, and psycho-oncology.

Furthermore, two additional late effects emerged as topics during some of the interviews and were also discussed in depth in those interviews. First, the fear of cancer recurrence was not believed to affect work ability by the interviewees but was considered important as it could impair mental resilience. Other studies reported that the fear of cancer recurrence can affect the quality of life [48], however, these studies were not focussed on work ability. Second, a different view of life was regarded as possibly influencing important choices regarding work. This change in the emotional meaning of work will likely be a result of a change in the perception of future time, as reported to occur in people of very different ages when experiencing illness, for instance [49]. Also attention was asked for post-traumatic growth [36], as cancer can also be regarded as a profound experience after which people may feel stronger mentally. However, for both late effects, no connection was seen with a lower work ability by the interviewees. So, guidance may be very important, but more from an approach that goes further than the effect on work ability now and in the near future.

In several healthy populations the lack of social support by colleagues is associated with feeling overwhelmed in the case of work problems [50]. Also the association of less social support by colleagues with lower work ability are reported [51]. As in healthy populations, also among workers past cancer diagnosis positive associations with work ability are reported in quantitative studies for social support by colleagues [21, 37, 52–55] or autonomy [54, 56, 57]. The interviewees in the present study regard a supporting role of colleagues as important for work ability and the exemplary behaviour of the manager in offering support as well. Furthermore, it is important to give the worker the opportunity to use as much autonomy as possible. However, it was reported that autonomy and self-leadership may be impaired because of the experience of getting cancer. In the case of an impaired ability of autonomous behaviour, it is possible to advise coaching or another form of guidance in personal development. Furthermore, there are individual differences between workers in the capability in self-leadership, and also companies differ significantly in the extent to which employees themselves can take control of their health [58]. In this respect, however, it was pointed out that certain interventions regarding for instance work load in the case of fatigue (for example, working fewer hours or another job with less demanding tasks), autonomy can be affected because the other tasks or job allows less autonomy. So different targets for interventions may work against each other, and the guidance therefore always should be a comprehensive package discussed between the worker, manager and professionals taking into account not only late effects but also the desired level of job resources. This means it is important not to focus only on a late effect, but also on other perspectives, especially concerning the available job resources.

Moreover, for this group of workers, an open culture or climate is a precondition for daring to come to the necessary communication to be able to ask for or receive guidance at all. It was raised that it is not always known in the work environment that a worker is confronted with late effects of cancer treatment. It may even be that it is completely unknown that the worker has had cancer. This can be for various reasons, but it can also be because people are afraid to tell, while in the case of late effects affecting work ability this would be the first step to be able to find solutions. Also, stigma may prevent the employee to talk openly about the work problems caused by disease or treatments, as is reported in the case of mental diseases [59, 60]. It is therefore very important for organizations to fight stigma and strive for a climate in which this openness does exist and is not dangerous. Moreover, a felt psychological safety is even of broader importance, as it has been shown to correlate with performance in general [61]. Unfortunately, the topic of organisational culture has not been studied previously in relation to workers coping with late effects of cancer treatments, to our knowledge. However, concerning organisational climate, which is a related concept [62], a positive relation of a better sociale climate at work with work ability was reported [63]. Regarding the guidance of workers past cancer diagnosis who experience late effects, the culture within an organization therefore may determine the possibilities to discuss a need for guidance. In general, it was emphasized that good communication with a worker who developed cancer a long time ago does not begin when late effects arise or appear to affect work ability. At the time of the diagnosis, open communication must already be possible and in fact it must even be present in the organization before that moment. In addition, of newly hired employees it may be completely unknown that they have had cancer in the past.

Finally, managers are not confronted with this population on a regular basis and their experience therefore is limited to a few specific cases at most. Consequently, although occupational physicians have knowledge and experience with this issue, in large organisations human resource management may build more expertise on this issue. However, in medium or small organisations this may be difficult and need more external input and resources. Therefore, other ways to share knowledge and ideas between organisations, for instance by human resource management associations or networks, can be of great importance. As far as rehabilitation is concerned, it is already clear that a multidisciplinary approach is needed [13], however the sharing of expertise and knowledge concerning the possible late effects of cancer (treatment) could be organised in a more interdisciplinary way. Also, interesting to hear during several interviews was, that certain professionals were also experts by experience. This had added an extra dimension to their professional guidance; knowledge and experience that may also be shared. Possibly there are specific ways to integrate these experiences into basic professional training as well, like suggested before by others regarding medical training and the experiences of illness and patienthood among general practitioners [64].

### Limitations

The interdisciplinary approach of this study, including not only health issues and clinical psychology, but also work psychology and organisational sciences, is a quite unique in studies regarding the long-term consequences of cancer (treatment) among workers. This study has highlighted therefore several important issues, however there are some limitations that should be noted.

First, it is important to emphasize that it is not clear to what extent the results are representative for all managers and within professional groups. Those who wanted to be interviewed for this study considered the topic important and furthermore relatively many had cancer themselves. It is not implausible that at least those who do not wish to pay attention to this topic did not participate. Hence, the positive attitude and the thinking in possibilities that emerged during the interviews therefore does not have to be the attitude among all other managers and professionals. However, since the aim was to explore ideas and possibilities, this is not a major problem. Furthermore, it is also essential to realize that the managers can have different levels of managerial experience, and the managers interviewed may differ from the average level of experience. Moreover, in everyday working life managers may also have a managerial position on a temporarily basis. Furthermore, certain professionals have not been interviewed at all. For example, no (applied) psychologists have been interviewed, while some of these professionals doubtless also discuss work with workers past cancer diagnosis coping with late effects of cancer or cancer treatment.

Second, we did not explicitly question the types of cancer and the time since diagnosis of the workers that the managers or professionals had experience discussing late effects with as we focused on the possible type of late effects with which they were confronted among workers. However, late effects may differ depending for instance on whether the cancer is within or outside the central nervous system, or because of surgery or radiation therapy affecting different sites. On the other hand, systemic therapies such as chemotherapy and endocrine therapy can be given in different types of cancer in different variants and may cause similar late effects across types of cancer. We can report that the experience of the managers and professionals involved many cases of workers with a past breast cancer diagnosis (a common cancer at working age with a high incidence and a high survival). However, we did not explicitly question the types of cancer and the time since diagnosis of the workers that the managers or professionals had experience with regarding any late effects affecting work ability. We focused on the possible type of late effects with which they were confronted among workers. However, it is possible that if a worker is many years after cancer diagnosis and experiences certain late effects for many years, the responses of the work environment or the possible solutions being considered may differ from the situations in which the period after cancer diagnosis is shorter.

Third, two specific groups of workers have been much less of a subject of discussion in this study, namely the self-employed and those who cope with recurrent or metastatic disease. Regarding the self-employed it is important to indicate that their situation is very different and more vulnerable in comparison with the salaried in several ways, financially and non-financially. As the self-employed do not have a manager and organisation that they can rely on for help and support, their options are limited. Nevertheless, in the present study mainly certain therapists, reintegration consultants and coaches reported also to guide non-salaried workers. Furthermore, the self-employed are a very diverse group of workers with different occupations and therefore different possibilities to generate solutions in the case of impairments or problems associated with late effects of cancer treatments. Nevertheless, studies report that the self-employed have a lot of worries, including concerns that the late effects of the treatments may continue to result in low work ability in the future [65]. Furthermore, in the present study no explicit attention was paid to those who have recurrent cancer or metastatic disease. However, workers who are affected by this are also among the workers who have had a cancer diagnosis in the past, but this subgroup obviously faces different challenges than those who assume or hope that they have been cured.

## Conclusions

This research entered a field about which little is known, namely late effects of cancer (treatment) among workers more than 2 years beyond cancer diagnosis and work ability. Completely new is the focus on the guidance of these workers by managers and professionals, in which both the late effects and the use of job resources play a role.

It has become clear that the interviewed managers and professionals observed a negative effect on work ability for the possible late physical effects, fatigue, or cognitive problems among this group of workers. In the case of physical late effects, the tailor-made solutions depend on the exact physical impairment(s) and the work tasks. Furthermore, in the case of fatigue guidance focuses on guarding boundaries, sufficient recovery time and guarding a good work-life balance. However, fatigue can be a complex problem, requiring in-depth examination and professional guidance. In view of the possible interrelations of fatigue with cognitive problems and possible confusion with burnout complaints, there are also many questions that require further research. In the case of cognitive probems there are ideas about practical actions for the guidance, like the planning of work tasks or a low-stimulus work environment. However, a need for more knowledge about the cognitive load of different ways of working and task structure was expressed. This also requires further research.

Regarding the job resources, there are no surprising results to report, but what appears to be very important is the need to be careful not to reduce job resources unnoticed when job changes are implemented to diminish impact of certain late effects. If that is the case (such as, for example, if someone were to get a less demanding job, with less autonomy at the same time), the result may be less positive than hoped for. This also underscores the importance of this study, as an interdisciplinary approach is proposed using the perspectives of health sciences, management, organizational psychology, clinical and neuropsychology.

Furthermore, an important contribution of this study is that it is not only focused on employees, but also on the self-employed. It has therefore become clear that the possibilities for guidance of the self-employed are not only more limited, because they are not part of an organization, but also that they receive guidance exclusively from certain professionals. However, also the employed seek additional guidance with these professionals, and it would be useful to investigate how the guidance by various professionals and managers combine with each other.

It is important that the way of dealing with late effects after cancer in the work environment, has to be positioned as a shared responsibility of the worker and their employer, as is argued regarding sustainable employability in general [66, 67]. Any late effects that affect work ability are not only a problem of the individual worker, but also of the organisation, as finding solutions requires a dialogue and must be a joint activity. Moreover, in some organizations there appears to be room for human resource management to fulfill a connecting role. It is also important to realize that on one hand there are still questions, but on the other hand a lot of knowledge and experience is available, including experiential knowledge. It is important to exchange more knowledge and experience between work organizations, professionals, including experiential knowledge, and share the insights also with the workers themselves.

To conclude, the long-term work ability after treatment for cancer needs an interdisciplinary approach, knowledge sharing and new knowledge gathering, and active involvement of the workers past cancer diagnoses confronted with late effects of cancer or cancer treatment themselves.

## Supplementary Information

**Additional file 1.** Interview Guide. Late effects of cancer (treatment) and work ability: guidance by managers and professionals.

**Additional file 2.** Research methodology. Late effects of cancer (treatment) and work ability: guidance by managers and professionals.

## Data Availability

The dataset analysed during the current study is not publicly available as it concerns the reports of the interviews, which can be traced back to individuals.

## Abbreviations

BACO: BedrijfsArts Consulent Oncologie (in Dutch)
In English: Occupational physician consultant oncology
cETO: Committee research EThics of the Open university
COVID-19: Corona VIrus Disease 2019
