# Supplementary material for: Late effects of cancer (treatment) and work ability: guidance by managers and professionals
Source: BMC Public Health. 2021 Jun 29;21:1255. doi: 10.1186/s12889-021-11261-2 (PMC8240423; doi:10.1186/s12889-021-11261-2)
Supplement: Supplementary file 1 — Additional file 1. Interview Guide. Late effects of cancer (treatment) and work ability: guidance by managers and professionals. [file 12889_2021_11261_MOESM1_ESM.docx]

**Additional File 1: Interview Guide**

**Late effects of cancer (treatment) and work ability: guidance by managers and professionals.**

| **Introduction** |
| --- |

*1. Thank you for this interview.*

*2. Introduction interviewer (role within this study and position).*

*3. Information regarding the backgrounds of the research line.*

This interview study is part of a research line that focusses on workers more than two years past cancer diagnosis. This focus is important because currently insufficient information is available about the group of workers who were diagnosed with cancer a long time ago. Most research is about the first two years after the diagnosis of cancer or about return to work. This research line is about the group of workers who are already two to ten years after diagnosis of cancer and have returned to work.

The aim is to clarify what is needed for this group to preserve or enhance work ability. Work ability can be described as the extent to which the worker physically, as well as mentally, is able to work, now and in the near future.

This information is important not only for those who work past cancer diagnosis, but also for their colleagues, managers, and professionals who guide these workers.

*3. About this interview study.*

These interviews are conducted with managers and professionals active in the field of guidance and support of (directly or indirectly) with people who work again after cancer. The interviews concern the ideas of the interviewee, about the experiences and possibilities in the approach in practice. The questions are not about specific individuals.

*4. Ask (once more) for permission for audio recording.*

*5. Give and have a letter of consent and a statement of consent read. Ask to sign (if not already read and signed).*

*6. Tell that a draft report will be e-mailed, to which the interviewee can respond (things different, in or out). In the case of remarks the report will be adjusted. Thereafter it will be anonymized.*

7. ***TURN ON AUDIO AND MENTION NAME INTERVIEWEE AND DATE!***

| **Interview Topics** |
| --- |

| **General** |
| --- |

Position (managerial or professional):

- Description of work tasks and professional responsibilities.
- How long have you been working in this position?
- Have you guided people who have had cancer directly?
- Collaboration with (other) managers or (other) professionals? Involved in case-management?

Organizational context:

- What attitude do you observe within the organizational context(s) towards absenteeism due to complaints and/or disorders?
- Impression of the degree of inflow and outflow of employees in general?
- How can the mutual contact between employees be described?
- Is cancer a point of attention within the organization? If so, to what extent?
- Use of specific guidelines regarding work and cancer?
- Is it usually known how workers with a past cancer diagnosis longer than two years ago are doing in the workplace? If so, how?

| **Late effects of cancer and cancer treatment and work ability** |
| --- |

- Are you familiar with these late effects of cancer and cancer treatment? Thoughts?
- What kind of late effects of cancer or cancer treatment have you noticed among workers?
  - Fatigue?
  - Cognitive (like problems with concentration)?
  - Physical?
  - Other?
- What is your view of the work ability of this group of workers?
- Is there any influence of late effects?
- What kind of bottlenecks do you observe among people after cancer in work? Barriers? Restrictions?
- With whom do the employees possibly share their experiences with possible late effects of cancer or cancer diagnosis within their organizational context?

| **Guidance?** |
| --- |

- Do you give advice regarding the content of the job or work tasks? If so, what do you advice in certain situations? Do you consult others? If so, who?
- What factors matter? What kind of guidance can be needed?
- Other activities from professional or managerial point of view?

| **Job resources?** |
| --- |

Introduction. Job resources are specific supporting factors in achieving work goals.

Possible job resources:

1. Autonomy; freedom; individual can make their own decisions within their work.

2. Support of colleagues.

3. Open organizational culture.

For each job resources questions in this line:

Do you observe the use of this job resource among workers past cancer diagnosis? If so, to what extent may this job resource help or can this help with the work ability in the case of late effects of cancer or cancer treatment? If not observed, what are your ideas about this job resource?

| **The worker with late effects** |
| --- |

- What can these workers do themselves to preserve or enhance their work ability? Regarding late effects? Regarding work tasks? Other?
- Does self-management or resilience affect any relationship between late effects and work ability?
- What could possibly enhance promote self-management or resilience among these workers?
- Additional: What would be sensible preventive measures in the group for whom the ability to work does not seem to be hindered by late effects?

| **Interventions?** |
| --- |

- To what extent can tasks and functions be customized? Willingness employers?
- What is being used in practice for interventions? Experiences?

| **Completion** |
| --- |

- Any other questions/comments/topics?
