# Supplementary material for: Late effects of cancer (treatment) and work ability: guidance by managers and professionals
Source: BMC Public Health. 2021 Jun 29;21:1255. doi: 10.1186/s12889-021-11261-2 (PMC8240423; doi:10.1186/s12889-021-11261-2)
Supplement: Supplementary file 2 — Additional file 2. Research methodology. Late effects of cancer (treatment) and work ability: guidance by managers and professionals. [file 12889_2021_11261_MOESM2_ESM.docx]

**Additional File 2: Research methodology.**

**Late effects of cancer (treatment) and work ability: guidance by managers and professionals.**

PLANNING DATA COLLECTION

- Recruitment of interviewees according to guidelines ethics committee.
- In case of a positive response: return mail with more general information and attached information letter and informed consent statement. Request to send a reply to make an appointment if interested. If so, an appointment was made. If not interested: a thank you. In the case of no reaction: one reminder after 1 or 2 months.

PREPARATION INTERVIEW GUIDE

- Determine and discuss thoroughly the aim of the project and interviews (all three authors).
- Prepare interview topics (using literature).
- Intensive involvement of the research trainees.
- Pilot interview.
- Adjustments interview guide.

DATA ANALYSIS

- Extract data from MAXQDA using the codes.
- Record frequencies of used codes, and the relevant texts by occupational role of the interviewee.
- Report results.

DATA CODING

- Directed content analysis: codes defined beforehand (using the interview guide) and during data analysis.
- Step 1:
  - Each research trainee coded three interviews, discussed this with the first author and the other research trainees.
  - New codes that emerged from the data were discussed and, if relevant, added in MAXQDA.
  - Any discrepancies were resolved through negotiated consensus.
- Step 2:
  - Coding of remaining interviews by a research trainee that was not present at the interview in question.
  - Check all coding interviews by first author and discrepant coding resolved by negotiated consensus.

DATA COLLECTION

- Interviews by first author and one of the research trainees.
- Location: work location interviewee, the Amsterdam University of Applied Sciences, or (video) calling (after lockdown because of COVID-19 pandemic always the latter).
- Start interview: information letter available, request to sign informed consent form. In the case of (video) calling, this was asked beforehand.
- Audio recording of the interview.
- Immediately after interview:
  - Audio file saved in secure data environment.
  - Audio file deleted from voice recorder.
  - Informed consent form scanned and in secure data environment.
- Draft interview report made by one of the interviewers and checked by the other. Draft report is sent to the interviewee within 2 – 3 weeks. The interviewee can make adjustments or additions, if desired.
- Anonymization final interview report (by using participant code).
- Upload final and anonymized interview report in MAXQDA.
